# Key genes associated with prognosis and metastasis of clear cell renal cell carcinoma

**DOI:** 10.7717/peerj.12493

**Published:** 2022-01-04

**Authors:** Tingting Zhong, Zeying Jiang, Xiangdong Wang, Honglei Wang, Meiyi Song, Wenfang Chen, Shicong Yang

**Affiliations:** Department of Pathology, The First Affiliated Hospital of Sun Yat-Sen University, Guangzhou, China

**Keywords:** Clear cell renal cell carcinoma, COL6A2, COL6A3, Metastasis, P4HB, PCOLCE

## Abstract

**Background:**

Clear cell renal cell carcinoma (ccRCC) is a tumor that frequently shows the hematogenous pathway and tends to be resistant to radiotherapy and chemotherapy. However, the exact mechanism of ccRCC metastasis remains unknown.

**Methods:**

Differentially expressed genes (DEGs) of three gene expression profiles (GSE85258, GSE105288 and GSE22541) downloaded from the Gene Expression Omnibus (GEO) database were analyzed by GEO2R analysis, and co-expressed DEGs among the datasets were identified using a Venn drawing tool. The co-expressed DEGs were investigated using Gene Ontology and Kyoto Encyclopedia of Genes and Genomes pathway enrichment analysis, and hub genes were determined based on the protein-protein interaction network established by STRING. After survival analysis performed on UALCAN website, possible key genes were selected and verified in ccRCC cell lines and ccRCC tissues (*n* = 44). Statistical analysis was conducted using GraphPad Prism (Version 8.1.1).

**Results:**

A total of 104 co-expressed DEGs were identified in the three datasets. Pathway analysis revealed that these genes were enriched in the extracellular matrix (ECM)–receptor interaction, protein digestion and absorption and focal adhesion. Survival analysis on 17 hub genes revealed that four key genes with a significant impact on survival: procollagen C-endopeptidase enhancer (*PCOLCE*), prolyl 4-hydroxylase subunit beta (*P4HB*), collagen type VI alpha 2 (*COL6A2*) and collagen type VI alpha 3 (*COL6A3*). Patients with higher expression of these key genes had worse survival than those with lower expression. *In vitro* experiments revealed that the mRNA expression levels of *PCOLCE*, *P4HB* and *COL6A2* were three times higher and that of *COL6A3* mRNA was 16 times higher in the metastatic ccRCC cell line Caki-1 than the corresponding primary cell line Caki-2. Immunohistochemistry revealed higher expression of the proteins encoded by these four genes in metastatic ccRCC compared with tumors from the corresponding primary sites, with statistical significance.

**Conclusion:**

PCOLCE, P4HB, COL6A2 and COL6A3 are upregulated in metastatic ccRCC and might be related to poor prognosis and distant metastases.

## Introduction

Clear cell renal cell carcinoma (ccRCC) is a highly aggressive malignancy that accounts for 85%–90% of all renal cell carcinoma cases (Chen et al., 2017). Approximately 17%–30% of ccRCC patients show distant metastasis at the time of diagnosis (Motzer, Bander & Nanus, 1996). Since ccRCC is not sensitive to radiotherapy and chemotherapy, radical or partial nephrectomy is still the main treatment, and 20% to 40% of cases that receive radical/partial nephrectomy still show recurrence or metastasis (Teng et al., 2014). Through the in-depth studies of ccRCC driving genes, studies have shown promising findings for molecular targeted treatments such as those targeting hypoxia-inducible factor 2α (*HIF2α*), indicating the potential success with new targeted therapies (Chen et al., 2016). However, treatments for patients with metastasis are still limited and the mechanism underlying ccRCC metastasis remains unclear. Hence, clarifying the mechanism on ccRCC metastasis is critical for of effective therapeutic targets to ccRCC patients with distant metastasis.

Microarray technology and bioinformatics analysis have facilitated the exploration of genetic alterations in various cancers. Bioinformatics analyses have helped researchers to identify genes specifically related with distant metastasis in a variety of tumors. For example, bioinformatics studies revealed that the expression of the sodium-potassium-chloride cotransporter 1 (NKCC1) gene was significantly higher in gastric cancer tissue than in normal gastric tissue. NKCC1 was shown to promote the proliferation, invasion, migration and epithelial to mesenchymal transition (EMT) of gastric cancer cells (Wang et al., 2021). Another study reported that U three protein 14a (UTP14A) stimulates the proliferation and metastasis of esophageal squamous cell carcinoma cells by regulating the PERK/eIF2a signaling pathway (Li et al., 2021).

With the aim to elucidate the key molecular events involved in metastatic ccRCC, we downloaded and analyzed three gene expression microarray datasets on ccRCC from GEO. We performed Gene Ontology (GO) and Kyoto Encyclopedia of Genes and Genomes (KEGG) enrichment of differentially expressed genes (DEGs) to investigate pathways involved in ccRCC metastasis. We also identified key factors associated with ccRCC prognosis and metastasis through protein-protein interaction (PPI) network construction, survival analysis and *in vitro* verification.

## Materials and Methods

### Microarray data collection

Three gene expression datasets, GSE85258 (Ho et al., 2017), GSE105288 (Nam et al., 2019) and GSE22541 (Wuttig et al., 2012), were selected and downloaded from GEO (http://www.ncbi.nlm.nih.gov/geo) (Edgar, Domrachev & Lash, 2002). GSE85258 includes 14 primary ccRCC samples and 14 patient-matched pulmonary metastatic ccRCC samples; GSE105288 includes nine primary ccRCC samples and 26 metastatic ccRCC samples; and GSE22541 includes 24 primary ccRCC samples and 24 pulmonary metastatic ccRCC samples. The samples above are from the primary and metastatic ccRCC sources. The GPL10558 Illumina HumanHT-12 V4.0 expression beadchip (GSE85258) and GPL570 [HG-U133_Plus_2] Affymetrix Human Genome U133 Plus 2.0 Array (GSE105288 and GSE22541) contain the microarray data of the three gene datasets.

### Identification of DEGs

GEO2R (http://www.ncbi.nlm.nih.gov/geo/geo2r), an interactive web tool for DEG analysis of GEO series across experimental conditions, was used to analyze the DEGs between primary ccRCC and metastatic ccRCC in the three datasets. To screen out more DEGs of these datasets, gene expression with —log2 fold change— > 0.5 and *P* < 0.05 were considered statistically significant. An online Venn drawing tool (http://bioinformatics.psb.ugent.be/webtools/Venn/) was used to obtain the intersecting co-expressed DEGs among the three datasets.

### Bioinformatic analyses of DEGs, hub gene selection and analysis

GO dataset (biological process (BP), cellular component (CC), molecular function (MF)) (Ashburner et al., 2000) and KEGG pathway (Kanehisa et al., 2010) functional enrichment analyses of DEGs were conducted by the Database for Annotation, Visualization and Integrated Discovery (DAVID, http://david.ncifcrf.gov/, version 6.8) (Dennis et al., 2003). False discovery rate (FDR) < 0.05 was considered statistically significant. The bubble plots of functional enrichment analysis were performed by bubble tools in Hiplot (https://hiplot.com.cn), a comprehensive web platform for scientific data visualization.

The Search Tool for the Retrieval of Interacting Genes (STRING, http://string-db.org, version 11.0) was used to predict the PPI network of DEGs (Szklarczyk et al., 2015). An interaction with a combined score > 0.4 among DEGs was recognized as statistical significance. The Molecular Complex Detection (MCODE) app of Cytoscape version 3.7.1 (Smoot et al., 2011) was used to cluster the PPI network of DEGs based on topology and to identify the closely connected area (Bandettini et al., 2012). The criteria were as follows: degree cut-off = 2, node score cut-off = 0.2, max depth = 100 and k-score = 2. A degree ≥ 10 was set for hub gene selection.

Survival analysis of hub genes was conducted on UALCAN (http://ualcan.path.uab.edu/index.html). *P* <0.01 was interpreted to be statistical significance to enroll crucial genes that are related to patients’ survival as many as possible. The UALCAN, which mainly based on the TCGA database, is also used to analyze the expression level of genes-of-shortlist between normal renal tissue and primary ccRCC. *P* < 0.05 was considered statistically significant. (Chandrashekar et al., 2017).

### Cell culture

Human ccRCC cell lines Caki-1 and Caki-2 were purchased from the BIOWING company in Shanghai. Cells were cultured in DMEM medium with 10% fetal bovine serum (FBS) and maintained at 37 °C in a 5% CO_2_ incubator.

### qRT-PCR

TRIzol was used to extract total RNA from cells. The Roche Transcriptor cDNA synthesis kit was used to reverse transcribe RNA to cDNA. qRT-PCR was performed on a Bio-Rad CFX-96 system (Bio-Rad), and reactions were performed in triplicate. The qRT-PCR primer sequences are as follows:

**Table:** 

Beta-Actin (F): 5′-CACCATTGGCAATGAGCGGTTC-3′
Beta-Actin (R): 5′- AGGTCTTTGCGGATGTCCACGT-3′
PCOLCE (F): 5′-GACACCTACTGCCGCTATGACT-3′
PCOLCE (R): 5′- GACGAGGAGTTCATTCCCTTCG-3′
P4HB (F): 5′-TCACCAAGGAGAACCTGCTGGA-3′
P4HB (R): 5′-GGCAAGAACAGCAGGATGTGAG-3′
COL6A2 (F): 5′-CGTGGAGACTCAGGACAGCCA-3′
COL6A2 (R): 5′-CCTTTCAAGCCAAAGTCGCCTC-3′
COL6A3 (F): 5′-CCTGGTGTAACTGATGCTGCCA-3′
COL6A3 (R): 5′-AAGATGGCGTCCACCTTGGACT-3′

### Immunohistochemistry

Paraffin-embedded formalin-fixed sections and clinical information of 22 paired primary ccRCCs and their metastatic counterparts were collected at the First Affiliated Hospital of Sun Yat-sen University. The metastatic sites included bone (10 cases), adrenal gland (3 cases), pancreas (2 cases), liver (2 cases), lung (1 case), brain (1 case), abdominal wall (1 case), nasal cavity (1 case) and chest wall (1 case). The study was approved by the institutional ethics committee of the First Affiliated Hospital of Sun Yat-sen University.

For section preparation, xylene and ethanol were used for deparaffinization and rehydration. The antigen retrieval procedure was performed with EDTA buffer in a microwave oven at 700 W for 5 minutes and then 600 W for 20 minutes. Slides were incubated in 3% hydrogen peroxide for 10 minutes and blocked in 5% bovine serum albumin (BSA) for 10 minutes. Slides were incubated with primary antibodies overnight at 4 °C. The primary antibodies included antibodies against PCOLCE (1:100; ab154261, Abcam), P4HB (1:200; ab137110, Abcam), COL6A2 (1:100; YT1035, Immunoway) and COL6A3 (1:100; YT1036, Immunoway). Slides were then incubated with SignalStain Boost IHC Detection Reagent (HRP, Rabbit, #8114, CST) as secondary antibody for 30 minutes at room temperature. The SignalStain DAB Substrate Kit (#8059, CST) was used for staining.

Slides were scanned with a Digital Pathological Section Scanner (KF-PRO-005) and three images were randomly selected in 200X magnification as the areas of interest (AOI), so as to minimize the effect of heterogeneity. The integral optical density (IOD) of each image was measured and calculated as the pixel area with Image Pro Plus for quantification of protein expression. Samples with a higher mean IOD value of AOIs were considered to have a stronger expression. After the quantification, 2 pathologists reviewed all slides and confirmed that the expression trends in the slides were consistent with the quantification results.

### Statistical analysis

Statistical analyses were conducted in GraphPad Prism (Version 8.1.1). The results of qRT-PCR were analyzed with unpaired *t*-test, and the results of immunohistochemistry quantification were analyzed with paired *t*-test. The error bars of qRT-PCR represent ± standard deviation (SD) of samples. *P* < 0.05 was considered statistically significant.

## Results

### Identification of DEGs in metastatic ccRCC

**Figure 1:**
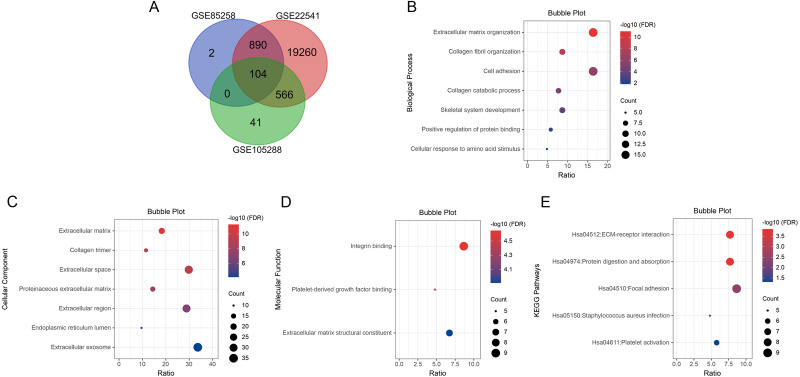
Venn diagram, GO enrichment analysis and KEGG pathway analysis (FDR < 0.05). (A) DEGs were selected with *P* < 0.05 and —log2 FoldChange— > 0.5 among the datasets GSE85258, GSE105288 and GSE22541. Venn diagram showed an overlap of 104 genes. (B) All BP results of 104 DEGs. (C) All CC results of 104 DEGs. (D) All MF results of 104 DEGs. (E) All KEGG pathway enrichment results of the 104 DEGs. The bubble plots were performed in Hiplot website.

We searched for DEGs in metastatic ccRCC samples compared with primary ccRCC samples in three datasets. A total of 996 DEGs in GSE85258, 711 DEGs in GSE105288 and 20,820 DEGs in GSE2254 were obtained. Venn diagram analysis revealed 104 co-expressed DEGs among the three datasets (Fig. 1A).

### Bioinformatic analyses of co-expressed DEGs

GO and KEGG pathway enrichment analyses were applied for the 104 DEGs using DAVID. The GO results revealed that in BP, DEGs were mainly enriched in extracellular matrix organization, collagen fibril organization, cell adhesion, collagen catabolic process, skeletal system development, positive regulation of protein binding, cellular response to amino acid stimulus and collagen biosynthetic process (Fig. 1B). In CC, DEGs were enriched in extracellular matrix, collagen trimer, extracellular space, proteinaceous extracellular matrix, extracellular region, endoplasmic reticulum lumen, extracellular exosome and basement membrane (Fig. 1C). In MF, DEGs were predominantly enriched in integrin binding, platelet-derived growth factor binding and extracellular matrix structural constituent (Fig. 1D). KEGG pathway analysis indicated that the DEGs were enriched in ECM-receptor interaction, protein digestion and absorption, focal adhesion, Staphylococcus aureus infection and platelet activation (Fig. 1E).

### PPI network construction, hub gene selection and key gene identification

**Figure 2:**
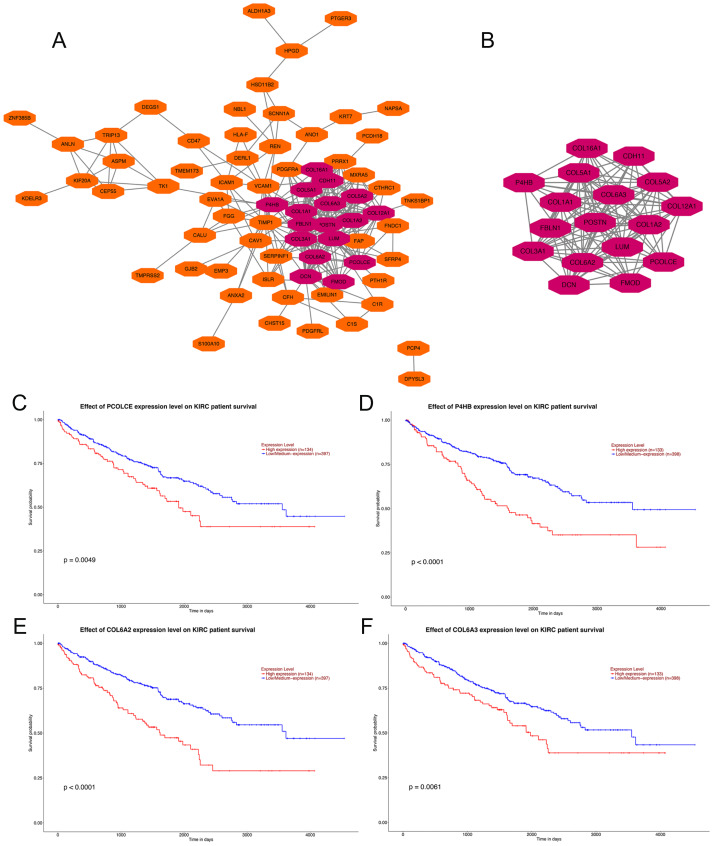
PPI network of DEGs, hub genes selection, and survival analysis of four key genes. (A) PPI network of 104 DEGs, the representation of nodes and edges are protein and the interaction of the proteins. (B) Seventeen hub genes of DEGs were obtained with module analysis using the MCODE app. (*P* < 0.01, KIRC: kidney renal clear cell carcinoma). (C) Survival analysis of *PCOLCE*. (D) Survival analysis of *P4HB*. (E) Survival analysis of *COL6A2*. (F) Survival analysis of *COL6A3*.

Analysis using the STRING online database demonstrated the PPI network of the DEGs. The most significant module containing 17 hub genes was established by the MCODE app of Cytoscape (Figs. 2A, 2B). GO and KEGG pathway enrichment analyses of the 17 hub genes are shown in Table 1. GO enrichment results revealed that the 17 hub genes were mainly enriched in extracellular matrix organization, collagen fibril organization, collagen catabolic process, skeletal system development cellular response to amino acid, cell adhesion and protein heterotrimerization in BP; extracellular matrix, collagen trimer, endoplasmic reticulum lumen, proteinaceous extracellular matrix, extracellular region 14, extracellular space and extracellular exosome in CC; and extracellular matrix structural constituent, platelet-derived growth factor binding, integrin binding, heparin binding, SMAD binding 3, collagen binding and peptidase activator activity in MF. KEGG pathways were mainly enriched in protein digestion and absorption, ECM-receptor interaction, focal adhesion, PI3K-Akt signaling pathway, Amoebiasis and Platelet activation (Table 1).

**Table 1:** GO and KEGG pathway enrichment analysis of 17 hub DEGs.

**Term**	**Description**	**Gene count**	**Gene ratio (%)**	**FDR**
**GOTERM_BP_DIRECT**				
GO:0030198	extracellular matrix organization	12	70.59	2.39E−16
GO:0030199	collagen fibril organization	8	47.06	1.62E−13
GO:0030574	collagen catabolic process	8	47.06	4.33E−12
GO:0001501	skeletal system development	7	41.18	6.92E−08
GO:0071230	cellular response to amino acid stimulus	5	29.41	2.35E−06
GO:0007155	cell adhesion	8	47.06	2.35E−06
GO:0070208	protein heterotrimerization	3	17.65	0.00154
GO:0043588	skin development	3	17.65	0.00821
GO:0001568	blood vessel development	3	17.65	0.00912
GO:0009612	response to mechanical stimulus	3	17.65	0.01975
GO:1903225	negative regulation of endodermal cell differentiation	2	11.76	0.02424
GO:0048592	eye morphogenesis	2	11.76	0.03332
GO:0007229	integrin-mediated signaling pathway	3	17.65	0.04214
**GOTERM_CC_DIRECT**				
GO:0031012	extracellular matrix	15	88.24	2.13E−22
GO:0005581	collagen trimer	9	52.94	5.36E−14
GO:0005788	endoplasmic reticulum lumen	10	58.82	1.33E−13
GO:0005578	proteinaceous extracellular matrix	10	58.82	2.06E−12
GO:0005576	extracellular region 14	14	82.35	4.64E−11
GO:0005615	extracellular space	12	70.59	4.99E−09
GO:0070062	extracellular exosome	10	58.82	7.93E−04
GO:1903561	extracellular vesicle	3	17.65	0.00302
GO:0005584	collagen type I trimer	2	11.76	0.00546
GO:0043202	lysosomal lumen	3	17.65	0.00614
GO:0005589	collagen type VI trimer	2	11.76	0.00614
GO:0005588	collagen type V trimer	2	11.76	0.00614
GO:0005796	Golgi lumen	3	17.65	0.00676
**GOTERM_MF_DIRECT**				
GO:0005201	extracellular matrix structural constituent	7	41.18	7.00E−10
GO:0048407	platelet-derived growth factor binding	4	23.53	1.66E−06
GO:0005178	integrin binding	5	29.41	2.35E−05
GO:0008201	heparin binding	4	23.53	0.00310
GO:0046332	SMAD binding 3	3	17.65	0.00431
GO:0005518	collagen binding	3	17.65	0.00698
GO:0016504	peptidase activator activity	2	11.76	0.03911
**KEGG_PATHWAY**				
hsa04974	Protein digestion and absorption	8	47.06	2.56E−11
hsa04512	ECM-receptor interaction	7	41.18	1.73E−09
hsa04510	Focal adhesion	7	41.18	2.12E−07
hsa04151	PI3K-Akt signaling pathway	7	41.18	3.37E−06
hsa05146	Amoebiasis	5	29.41	1.04E−05
hsa04611	Platelet activation	5	29.41	2.34E−05

**Notes.**

GO Gene Ontology MF Molecular Function CC cellular component BP biological process KEGG Kyoto Encyclopedia of Genes and Genomes

Four genes, *PCOLCE*, *P4HB*, *COL6A2* and *COL6A3* were sorted out with *P*-value < 0.01 among the 17 hub genes after survival analysis performed on the UALCAN website. Data showed that patients with high expression of *PCOLCE*, *P4HB*, *COL6A2* and *COL6A3* had worse survival than those with low expression of each gene (Figs. 2A, 2B). Also, these four genes were found to be higher expressed in primary ccRCC compared to normal kidney tissue (Fig. S1).

### *In vitro* validation of the four key genes in metastatic ccRCC

**Figure 3:**
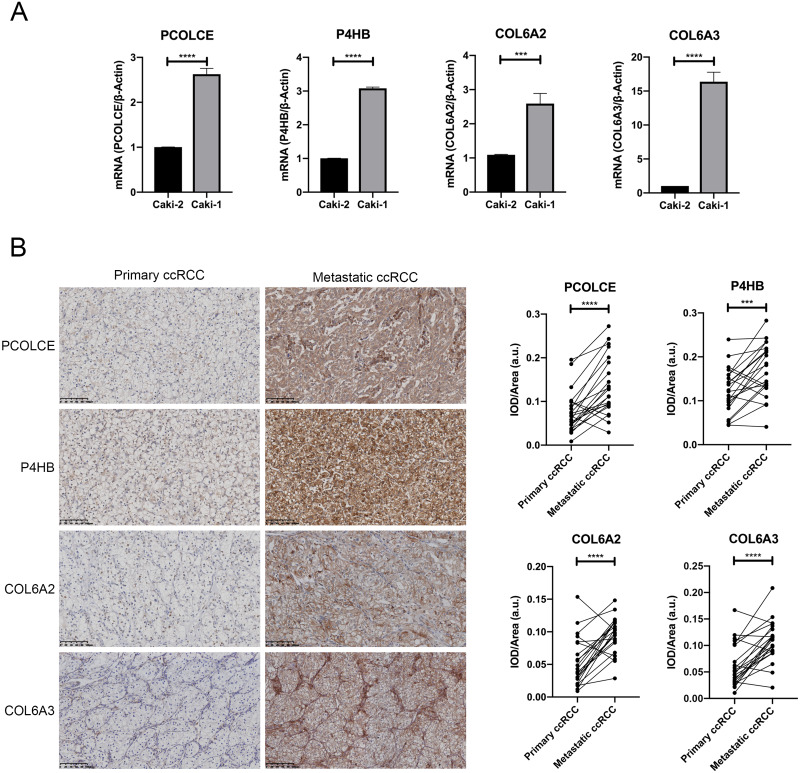
Expression of key genes in primary ccRCC and the corresponding metastatic ccRCC. (A) the qRT-PCR analysis showed the expression of *PCOLCE*, *P4HB*, *COL6A2* and *COL6A3* in primary ccRCC cell line Caki-2 and its corresponding metastatic ccRCC cell line Caki-1. (^∗∗∗^*P* < 0.001, ^∗∗∗∗^*P* < 0.0001) (B) Representative IHC staining images of *PCOLCE*, *P4HB*, *COL6A2* and *COL6A3* in human primary ccRCC and corresponding metastatic ccRCC (22 paired cases) are on the left, and the quantitative results on the right. 200x. (^∗∗∗^*P* < 0.001, ^∗∗∗∗^*P* < 0.0001).

qRT-PCR showed that *PCOLCE*, *P4HB*, *COL6A2* and *COL6A3* mRNAs were all upregulated in the Caki-1 cell line compared with its primary counterpart, the Caki-2 cell line (*P* < 0.05) (Fig. 3A).

We further examined the expressions of the proteins encoded by the four genes in 22 pairs of primary ccRCC and their corresponding metastatic counterpart tissue with immunohistochemistry. The TNM staging information of 12 cases of primary ccRCC at first diagnosis is presented in Table S1. Higher expressions of all four proteins were observed in most metastatic ccRCC tissues compared with the primary sites, as shown by mean IOD results (*P* < 0.05). Higher expressions of COL6A2 and PCOLCE in metastatic counterparts were observed in 19 out of 22 pairs, and higher expressions of COL6A3 and P4HB expression were detected in 17 out of 22 pairs (Fig. 3B).

## Discussion

CcRCC is believed to originate from proximal tubular epithelium with aggressive behavior that was unparallel with its bland appearance. After surgery, the median survival time of ccRCC patients with metastasis is only 13 months, and the 5-year survival rate is 12.3% (Babaian et al., 2014). As one of the main genetic events contributing to ccRCC pathogenesis, the mutant *VHL* gene pathway has become a target for treatment towards ccRCC (Chen et al., 2016). With the ongoing effort in bioinformatic data management, novel candidate key genes may be identified as new therapeutic targets for ccRCC patients with metastasis. In order to obtain datasets with a long time span and sufficient samples from different institutions, we downloaded 3 datasets from GEO websites to screen out the intersection of differential expression genes.

In our study, we first obtained 104 co-expressed DEGs from three datasets. In the course of searching for key genes involved in ccRCC metastasis, we conducted bioinformatic analysis on 17 hub genes which were screened from the PPI network analysis to identify specific pathways that might be related to ccRCC metastasis. GO analysis showed the DEGs were mainly distributed in extracellular matrix, extracellular space, extracellular exosome and Golgi lumen of CC, and were mainly enriched in extracellular matrix organization, collagen fibril organization, cell adhesion and blood vessel development of BP, and extracellular matrix structural constituents, platelet-derived growth factor binding, integrin binding and collagen binding of MF. KEGG pathway analysis revealed that these DEGs were enriched in protein digestion and absorption, ECM-receptor interaction, focal adhesion and PI3K-Akt signaling pathway.

Exosomes, which functioned in carrying protein, ceramide, mRNA or micro-RNA as cargo, can induce metastasis through participation in EMT, mesenchymal-epithelial reverting transition and neoangiogenesis (Steinbichler et al., 2017). For example, in ccRCC cell lines, exosomes containing miR-19b-3p could initiate EMT and accelerate tumor metastasis by reducing PTEN expression (Wang et al., 2019a; Wang et al., 2019b). EMT was essential for tumor metastasis through inducing processes such as loss of cell polarity, disconnection with the basement membrane, acquiring plasticity, invasion and anti-apoptosis activities (Talbot, Bhattacharya & Kuo, 2012). Additionally, cell adhesion to ECM participates in multiple steps of tumor metastasis through ECM receptor interaction (Hamidi & Ivaska, 2018). For example, through the several binding sites from matricellular proteins members of the CCN family, integrin could help tumor cells resist anoikis and promote tumor cells metastasize (Lau, 2016; Simpson, Anyiwe & Schimmer, 2008). Studies have shown that the PI3K-Akt signaling pathway might induce the EMT process directly or through crosstalk with the Wnt/β-catenin signaling pathway, TGF-β or NF-kB and initiate distant metastasis (Xu, Yang & Lu, 2015). The phosphorylated AKT, a key kinase of the PI3K signaling pathway, was reported to be related to EMT in ccRCC (Wu et al., 2011). Our results also showed that DEGs in metastatic ccRCC were enriched in the PI3K signaling pathway, suggesting that PI3K signaling pathway–mediated EMT was likely to participate in the metastasis of ccRCC. Together, our results have revealed various processes that might be involved in ccRCC metastasis.

To explore the relationship between 17 hub genes and patient survival, the survival analyses were conducted. From survival analyses, *PCOLCE*, *P4HB*, *COL6A2* and *COL6A3* were found to be related to ccRCC patient survival, and these candidate genes were further tested with qRT-PCR and immunohistochemical staining in ccRCC cell lines and tissues. We found that the four genes showed higher expression in the metastatic counterpart of ccRCC both in cell lines and paired ccRCC tissues. *P4HB* and *COL6A3* have been previously reported to be associated with ccRCC. And our study presents the linkage of *PCOLCE* and *COL6A2* with ccRCC.

To the best of our knowledge, this is the first report that showed the relevance between *PCOLCE*and ccRCC. PCOLCE is a secreted glycoprotein that participates in the maturation of procollagen and ECM reconstruction by enhancing the activity of bone morphogenetic protein-1 (BMP-1) (Kessler, Mould & Hulmes, 1990; Moali et al., 2005; Zhu et al., 2019a; Zhu et al., 2019b). *PCOLCE* was overexpressed in osteosarcoma and promoted lung metastasis via twist family bHLH transcription factor 1 (TWIST1). Reducing *PCOLCE* expression could prevent the migration, invasion and lung metastasis of osteosarcoma cells (Wang et al., 2019a; Wang et al., 2019b). *BMP-1* was reported to be up-regulated in gastric cancer and associated with poor survival and distant metastasis (Hsieh et al., 2018). Notably, a previous study showed that BMP-1 was elevated in ccRCC and promoted proliferation, migration and invasion in ccRCC cell lines (Xiao et al., 2020). Our study showed that the expression of *PCOLCE*, which functions as an enhancer of *BMP-1*, was higher in metastatic ccRCC. This finding raised the hypothesis that PCOLCE might be involved in ccRCC metastasis through regulation of *BMP-1*. Therefore, further research is needed to clarify the function and potential mechanism of PCOLCE in ccRCC metastasis.

P4HB, a beta subunit of prolyl 4-hydroxylase, helps cancer cells survive from apoptosis induced by endoplasmic reticulum stress (Lovat et al., 2008; Pajunen et al., 1991). P4HB promoted proliferation, invasion, migration and angiogenesis in glioma through the mitogen-activated protein kinase (MAPK) signaling pathway (Sun et al., 2017). In gastric cancer, P4HB was shown to play an important role in regulating invasion and migration in the HIF1*α* pathway (Zhang et al., 2019). Previous studies showed that HIF1*α* was activated in renal clear cell carcinoma and associated with a more aggressive phenotype (Maxwell et al., 1999; Na et al., 2003). And the overexpression of *P4HB* in ccRCC was associated with poor outcome (Zhu et al., 2019a; Zhu et al., 2019b). In our study, we found that *P4HB* was specifically elevated in metastatic ccRCC. Hence, these findings suggest that P4HB might play a key role in modulating ccRCC metastasis through HIF1*α* pathway.

In this study, we showed that *COL6A2* and *COL6A3* were related to ccRCC progression. COL6A1–3 are three subunits of collagen VI (Hessle & Engvall, 1984; Weil et al., 1988). Strong evidence has demonstrated the role of COL6A1–3 in promoting the progression of tumors (Chen, Cescon & Bonaldo, 2013). In serous ovarian cancer, *COL6A2* was dysregulated and associated with poor prognosis through the TGF*β*1 pathway (Cheon et al., 2014; Svoronos et al., 2020). COL6A3 functions in tumorigenesis and progression of cholangiocarcinoma through the E2F1/LMCD1-AS1/miR-345-5p/COL6A3 axis and serves as prognostic factor for pancreatic cancer (Yu et al., 2019). Up-regulation of *COL6A1* in ccRCC was associated with poor prognosis of patients and promoted tumor growth *in vivo* (Wan et al., 2015). *COL6A3* was reported to be overexpressed in metastatic renal cell carcinoma and associated with poor survival (Li et al., 2019). To date, no report has described the relation between *COL6A2* and ccRCC. As *COL6A2* and *COL6A3* were identified as key genes in our results, further research should be carried out to examine their mechanisms in ccRCC metastasis.

This study has several limitations. First, we only examined 22 paired ccRCC samples and 2 cell lines to verify the bio-informatic findings. More samples need to be examined to confirm our results. Second, the mechanisms of the four key genes in regulating ccRCC cell metastasis are still unclear. Further exploration is required to clarify the function and potential mechanism of the four key proteins both *in vitro* and *in vivo*.

## Conclusion

Our studies identified four key genes (*PCOLCE*, *P4HB*, *COL6A2* and *COL6A3*) that were related to ccRCC patient survival and showed potential involvement in ccRCC metastasis. The proteins encoded by the four genes may be involved in the ECM-receptor interaction, focal adhesion and the PI3K-Akt signaling pathway. In vitro experiments confirmed increased expression of the candidate genes in metastatic ccRCC, both in cell lines and tissues. Future studies are required to evaluate the significance and mechanism of these key proteins in ccRCC.

## Supplemental Information

10.7717/peerj.12493/supp-1 Supplemental Information 1 The expression levels of the 4 key genes which are based on the TCGA database (A) The expression level of PCOLCE in normal and KIRC samples. (B) The expression level of P4HB in normal and KIRC samples. (C) The expression level of COL6A2 in normal and KIRC samples. (D) The expression level of COL6A3 in normal and KIRC samples. *P* < 0.05 was considered statistically significant.

10.7717/peerj.12493/supp-2 Supplemental Information 2 Clinical information and TNM staging of ccRCC cases

10.7717/peerj.12493/supp-3 Supplemental Information 3 Raw data qRT-PCR & semi-quantitative immunohistochemistry results

## Additional Information and Declarations

### Data Availability

The following information was supplied regarding data availability: The raw measurements are available in the Supplementary File.
