# Supplementary figures and images for: Key genes associated with prognosis and metastasis of clear cell renal cell carcinoma
Source: PeerJ. 2022 Jan 4;10:e12493. doi: 10.7717/peerj.12493 (PMC8740509; doi:10.7717/peerj.12493)

A

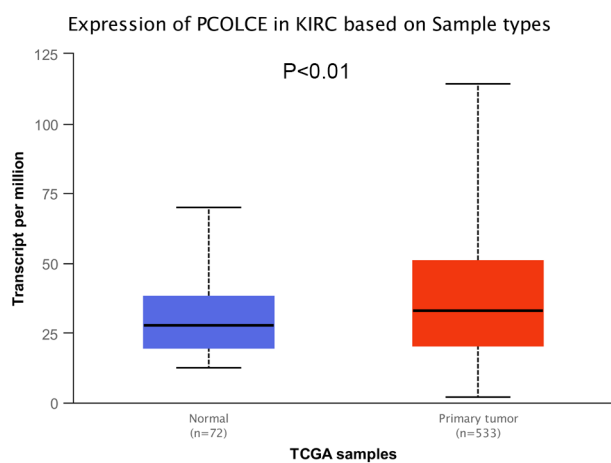

B

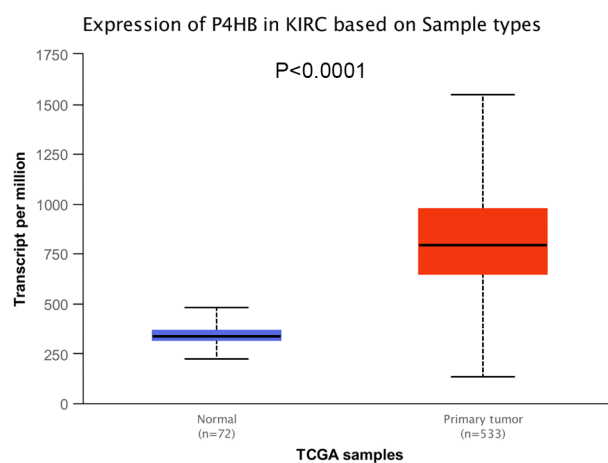

C

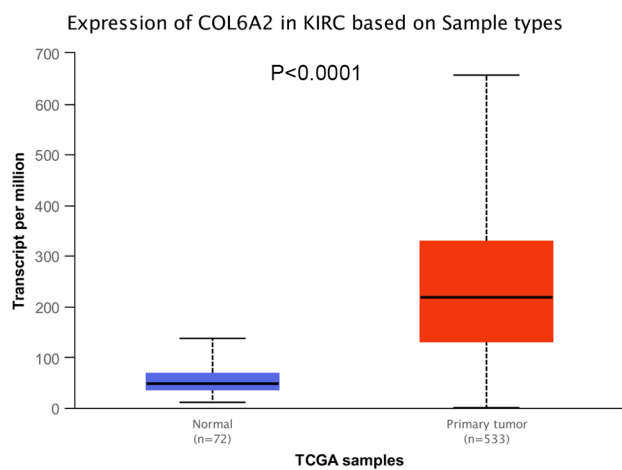

D

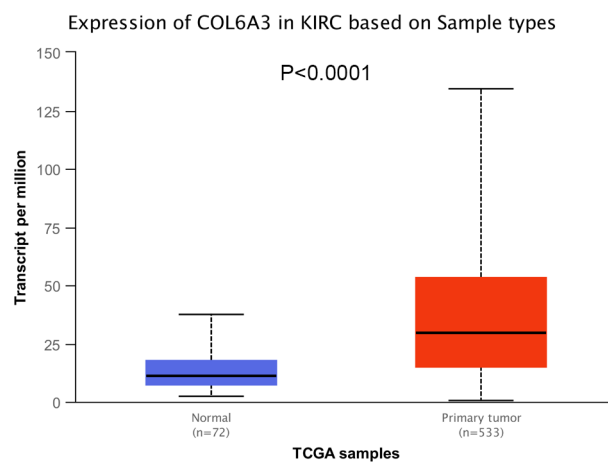

Supplement: Supplemental Information 1 — (A) The expression level of PCOLCE in normal and KIRC samples. (B) The expression level of P4HB in normal and KIRC samples. (C) The expression level of COL6A2 in normal and KIRC samples. (D) The expression level of COL6A3 in normal and KIRC samples. P < 0.05 was considered statistically significant. [file peerj-10-12493-s001.pdf]
