# Supplementary material for: Key genes associated with prognosis and metastasis of clear cell renal cell carcinoma
Source: PeerJ. 2022 Jan 4;10:e12493. doi: 10.7717/peerj.12493 (PMC8740509; doi:10.7717/peerj.12493)
Supplement: Supplemental Information 2 [file peerj-10-12493-s002.docx]

| Case  (primary ccRCC) | Volume of the tumor (cm) | Lymph node (LN) metastasis | Distant metastasis | TNM stage (first-diagnosis) | Corresponding metastatic sites |
| --- | --- | --- | --- | --- | --- |
| 1 | 3$\times$2.5 | No enlarged LN | No metastasis | T1a N0 M0 | Liver |
| 2 | 7$\times$5.2$\times$3 | No enlarged retroperitoneal LN | Caudal pancreas  metastasis | T1b N0 M1 | Pancreas |
| 3 | 5 (diameter) | No enlarged retroperitoneal LN | The boundary between the tumor and the left psoas major and quadratus lumborum is not clear | T4 N0 M0 | Bone |
| 4 | 8 (diameter) | Several mildly enlarged LN in the mesenteric area of the right lower abdomen, but not in the rest of the body. | Contralateral adrenal metastasis | T2a N0 M1 | Adrenal gland |
| 5 | 4.2$\times$3$\times$3 | No enlarged LN | Thoracic vertebrae (T12) metastasis | T1b N0 M1 | Bone |
| 6 | 7.5 (diameter) | Retroperitoneal LN metastasis | Scapula metastasis | T3a N1 M1 | Bone |
| 7 | 4$\times$3.5$\times$2.5 | No enlarged retroperitoneal LN | Cancer embolus in renal portal system | T3a N0 M0 | Bone |
| 8 | 2.5 (diameter) | No enlarged LN | Proximal femur metastasis | T3 N0 M1 | Bone |
| 9 | 10$\times$8$\times$7 | Mildly enlarged retroperitoneal LN | Chest wall metastasis | T2a N0 M1 | Chest wall |
| 10 | 6 (diameter) | No enlarged LN | No metastasis | T1b N0 M0 | Bone |
| 11 | 3 (diameter) | No enlarged LN | No metastasis | T1a N0 M0 | Bone |
| 12 | 4$\times$5 | No enlarged LN | No metastasis | T1 N0 M0 | Bone |
| 13 | 3 (diameter) | NA | NA | NA | Bone |
| 14 | 5$\times$4$\times$6 | NA | NA | NA | Bone |
| 15 | 4 (diameter) | NA | NA | NA | Bone |
| 16 | 7$\times$4.5$\times$4 | No metastatic LN in renal hilus | NA | NA | Brain |
| 17 | 10 (diameter) | No enlarged LN | NA | NA | Adrenal gland |
| 18 | 4 (diameter) | No enlarged LN near renal artery or abdominal aorta | NA | NA | Adrenal gland |
| 19 | 5 (diameter) | NA | NA | NA | Nasal cavity |
| 20 | 6 (diameter) | No metastatic LN in renal hilus | NA | NA | Abdominal wall |
| 21 | 11$\times$7$\times$6 | NA | NA | NA | Lung |
| 22 | 5 (diameter) | Para-aortic and renal hilus LN metastasis | NA | NA | Liver |

Clinical information and TNM staging of ccRCC cases:
